# Sleep Disordered Breathing: An Epidemiological Study among Albanian Children and Adolescents

**DOI:** 10.3390/ijerph17228586

**Published:** 2020-11-19

**Authors:** Yllka Abazi, Fabian Cenko, Marianna Cardella, Gjergji Tafa, Giuseppina Laganà

**Affiliations:** 1 Department of Biomedical Science, Catholic University Our Lady of Good Counsel, 1000 Tirana, Albania; f.cenko@unizkm.al; 2 Private Practice, 00100 Rome, Italy; cardella.marianna@gmail.com; 3 Private Practice, 1000 Tirana, Albania; gjergjitafa12@gmail.com; 4 Department of Clinical Sciences and Translational Medicine, University Tor Vergata, 00133 Rome, Italy; giuseppinalagana@libero.it

**Keywords:** SDB, questionnaire PSQ-SRDB, Albanian population

## Abstract

Sleep Disordered Breathing (SDB) comprises a group of diseases characterized by alterations in the frequency and/or depth of breathing during sleep. The aim of this study was to investigate the frequency of SDB in a group of Albanian children and adolescents and to describe its social, physiological, psychological, sleep-related, and anthropometric risk factors, in relation to the sociodemographic situation. A total of 6087 participants (mean age: 10.42 years, range: 6 to 15 years, 52.3% females and 47.7% males) attending public schools all over Albania took part in the cross-sectional study. On a sample of 6087 questionnaires distributed, 4702 (77.25% of the original sample) were returned and included in the study. High risk status for SDB was assessed using the Paediatric Sleep Questionnaire (PSQ). The prevalence of SDB was 7.9%. No statistically significant difference was found for gender at high risk for SBD. Compared to participants living in urban aeras (7.3%), participants living in rural areas (10.4%) reported significantly higher SDB prevalence rates. No other significant correlations were detected between the high-risk subjects and the age. The prevalence of the subjects at high risk of SBD obese participants (20.8%) was statistically higher than among nonobese ones (6.3%). SDB is highly prevalent in Albanian growing population and further prevalence studies are recommended.

## 1. Introduction

Sleep Disordered Breathing (SDB) comprises a group of diseases ranging from snoring to Obstructive Sleep Apnea (OSA), characterized by alterations in the frequency and/or depth of breathing during sleep [1]. SDB in childhood can lead to behavioral and cognitive impairments that might be related to the particular vulnerability of the pre-frontal cortex [2]. The pediatric individuals with sleep disorders present hyperactivity, restlessness, lack of attention, loss of appetite and irritability [3,4]. If left untreated, SDB in development age could be responsible to significant morbidity leading to metabolic syndrome [5], cardiovascular consequences [6], and growth failure [7]. The negative impact of SDB may not simply be confined to the short-term wellness and development during childhood but may continue adversely affecting during the long-term development in adulthood.

There are many risk factors that can lead to a reduction or collapse of upper airways and which may contribute to the pathogenesis of SDB. Pediatric sleep disorders are associated with at least four clinical phenotypes: Adenotonsillar hypertrophy, orthodontic and craniofacial alterations and syndromic conditions, primary neuromuscular disorders, and obesity. The emerging epidemic of obesity has refocused attention to this phenotype today. However, the relationship between obesity and SDB in children and adolescent is clearly conflicting.

Despite increased awareness in the adult population, SDB often goes undetected in childhood and in adolescent age. The clinical presentation of a child with SDB is usually nonspecific, requiring increased expertise of the primary care professional. The prevalence of this disorder differs from 1 to 5.8% in pediatric patients, referring to studies using accurately and objective diagnostic measures such as polysomnography and pulse oximetry [1,8,9], and 4–11% based on questionnaire surveys of parents [10,11].

The gold standard diagnosis consists in polysomnography (PSG), although its utilization is not very common because it requires a “sleep medical center”, which implies long time, qualified operators, high-tech devices, and high costs related to these conditions. A first step would be carrying on epidemiological studies in order to assess the prevalence of individuals at high risk utilizing specific self-administered questionnaires [12].

The aim of this study is to determine, in growing Albanian populations, the prevalence levels of SDB as a function of sex, age group, obesity, and urban/rural residence categories, and its social, physiological, psychological, sleep-related, and anthropometric risk factors. Yet, there are no reports on SDB of Albanian children and adolescents and its association with their sociodemographic factors.

## 2. Materials and Methods

### 2.1. Study Population

The target population of this cross-sectional study was composed of growing subjects; the sample covered a random sample of public schools in the largest areas of the country. The selected sample was chosen during the period October 2018–May 2019.

A special permission issued from Ministry of Education Sport and Youth (MESY) of Albania regulated the data collection. Through the MESY, the data about the total number of students in public schools for the 2018–2019 school year was obtained. A two-stage sampling strategy was applied in order to ensure the representativeness of the sample for all students attending the obligatory public schools in the country. The first stage referring to a random selection of the schools, and the second stage sampling procedure selecting the classes inside each school. The total number of participants required was proportional to the population density by district. The final sample was composed of 6087 subjects between the ages of 6 and 15, representing 4.24% of the public-school population in Albania.

In each selected school, during the first stage, the survey team delivered the relative questionnaires to the selected classes from the second stage. Then, inside each selected class, a questionnaire was handed over to attending students in order to be delivered and completed from their parents.

**Figure 1:**
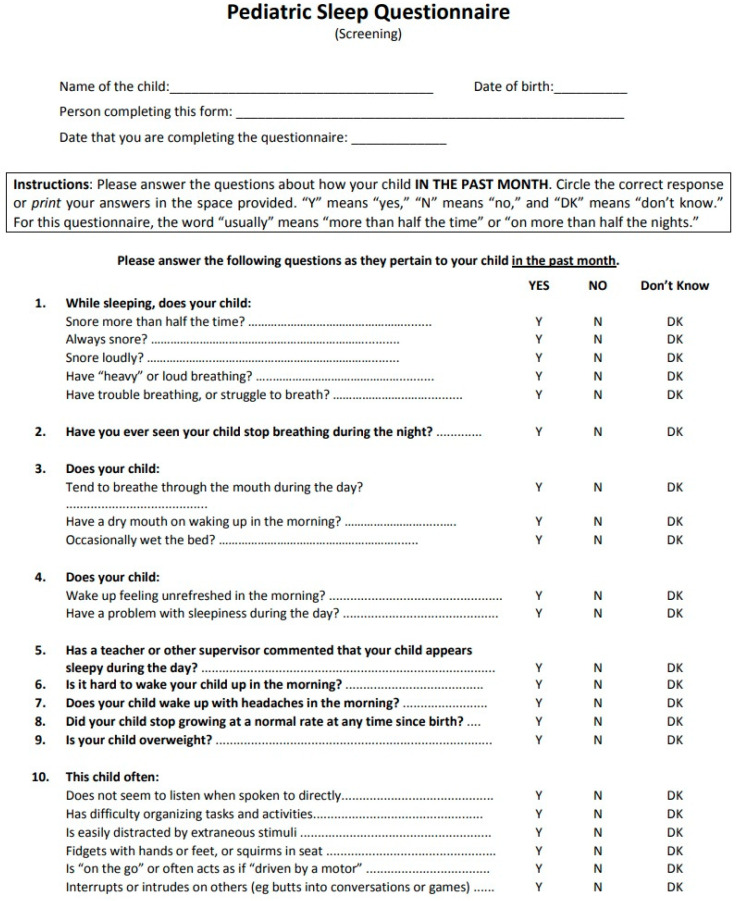
Pediatric Sleep Questionnaire.

The questionnaire can be simplified into two parts: the first page contains demographic information (age, gender, residence) and consensus confirmation signed from the parents, respecting the privacy of personal data according to the Helsinki Declaration principles. Two trained students (Marianna Cardella and Gjergji Tafa) collected anthropometric measurements from the subjects’ height (m) and weight (kg) using a digital scale. The BMI (kg/m^2^) was calculated and overweight/obesity was defined as ≥ 25 kg/m^2^ [13]. On the second page, the 22 closed-response questions consisted of prominent symptoms of sleep disorders. The following specific criteria for exclusion have been verified for each participant before added in the final sample: previous or current orthodontic therapy, syndromes and compromised craniofacial anomalies (Figure 1). The filled and signed questionnaires were collected the next day of the delivery.

The whole process of questionnaire distribution and data collection was carried out from the same team composed from two students and one professor from Faculty of Dentistry of the Catholic University of “Our Lady of Good Counsel” in Tirana, Albania. The same University financially sustained this epidemiological study.

This study followed the principles laid down by the World Medical Assembly in the Declaration of Helsinki 2008 Helsinki Declarations on medical protocols and ethics and received positive response by the Ethic Committee at the University of Rome Tor Vergata (n° 139/2019).

### 2.2. The Definition of PSQ-SDRB Scale

Specific self-administered questionnaires could be effectively utilized to diagnose SDB and evaluate the progress of treatment. This process requires utilization of validated prospective Pediatric Sleep Questionnaire (PSQ), developed for individuals from 2 to 18 years old assessing the presence of specific symptoms such as snoring, apneas, sleepiness, and behavior disorders. The Pediatric Sleep Questionnaire, originally published by Chervin et al., yielded better results than other published questionnaires, reaching the sensitivity as high as 85% and specificity level as high as 87% [4].

The Sleep-Related Breathing Disorders (SRDB) scale contains 22 symptoms indicating snoring frequency, noise, observed apnea, difficulty breathing during sleep, daytime sleepiness, unreliable or hyperactive behavior, and other pediatric SDB characteristics, each of them previously correlated with polysomnography confirmed OSA in affected growing individuals. The scale score for each individual questionnaire is done by dividing the total number of questions answered in the affirmative (YES) by the number of questions answered both in an affirmative (YES) and negative (NO). In this formula has been excluded the questions with “don’t know” or “don’t reply” response from the numerator and denominator. The result, which is a number ranging from 0.0 to 1.0, when scoring > 0.33 is considered positive and suggests a high risk for SDB in participants [14]. Otherwise, scale score ≤ 0.33, define a person not at risk for SDB. The subscales within the SRDB scale include a 4-element sleepiness scale, a 4-item snoring scale, and a 6-element inattention/hyperactivity scale element originally derived from the Diagnostic and Statistical Manual of Mental Disorders (DSM-IV) and criteria for attention-deficit/hyperactivity (ADHD).

Before proceeding with the translation of the questionnaire, an official request was sent to the Author to obtain the authorization for the translation in respect to the Copyright. After having obtained the authorization, preserved in the archives of the Catholic University Our Lady of Good Council in Tirana and the University of Michigan, the subsequent phases of linguistic validation was performed [15]. This process was recommended by Beaton et al. [16], including the following steps:

1- Forward translation of the questionnaire from English into Albanian by two translators;
2- Synthesis of the two translations;
3- Back-translation;
4- Evaluation by an expert committee;
5- Pretesting of the penultimate version.

The direction of validation process was entrusted to a single project manager with the task of checking, correcting and managing the different phases.

### 2.3. Statistical Analysis

All data were analyzed with the Statistical Package for the Social Sciences software (SPSS^®^ 26.0 (IBM Corporation, Armonk NY, USA) for Apple Mac^®^). Internal consistency and item-total score correlations were used for reliability analysis. Internal consistency was tested using Cronbach alpha for every sub-scale of the instrument since every sub-scale represents a different concept for the patients. Cronbach’s α values less than 0.50 were regarded as not acceptable. The item score and total score relationships were explored by Pearson’s correlation analysis. Validity of the Albanian version of the PSQ was tested by construct validity analysis. Construct validity was tested by using known group method tested by ANOVA analysis.

Descriptive statistics are presented (means and standard deviations or percentages) for quantitative and qualitative variables, respectively. PSQ scores were examined by sex, age, and region-based categories, and confidence intervals have been calculated according to Normal Approximation to the Binomial according to the Standard Normal Distribution. Correlations of PSQ scores with age were evaluated with the Spearman rank correlation coefficient (rho). The linear regression for bivariable analyses was applied using the Student *t* distribution and *F* distribution.

## 3. Results

On a sample of 6087 questionnaires distributed, there was a return of 4702 (77.25% of the original sample). Out of 4702 filled-in questionnaires returned, 4442 were considered appropriately completed (94.47%); 260 questionnaires were excluded cause of not answering all the questions (5.53%).

According to the division of the sample based on urban and rural residence, the urban sample was larger than the rural areas one (83% versus 17%): only in Tirana and Durresi, due to logistical, timing and economic issues, the sample was distributed in both residences. Regarding the district-based distribution, the subsamples from each district are not equal because they are proportional to the surveyed target population. According to INSTAT [17], Tirana represents almost half of the total sample based on its general population about one third of the whole country.

Table 1 shows the Cronbach’s alpha value for snoring domain, which was 0.63 (4 items/questions). Item scale correlations for snoring domain were significant only for the questions “Does your child snore loudly” and “Does your child always snore” where the relative correlation coefficients were 0.53 and 0.51 accordingly. The remaining two questions “Does your child snore more than half the time” and “Does your child have heavy or loud breathing” the correlation coefficients were 0.32 and 0.34, respectively.

For sleepiness domain, Cronbach’s alpha value was 0.38. None of the singular questions were significant such as “does your child wake up feeling unrefreshed in the morning”, “Does your child have a problem with sleepiness during the day”, “Has a teacher o other supervisor commented your child appears sleepy during the day”, and “Is it hard to wake your child up in the morning” had the following respective correlation coefficients 0.27, 0.27, 0.07, and 0.21, respectively.

Cronbach’s alpha value for inattention domain was 0.53. Item scale correlations for the items “does not seem to listen when spoken directly”, “Has difficulty organizing tasks and activities” and “Is easily distracted by extraneous stimuli” were 0.29, 0.25, and 0.28, respectively. Similarly, items “Fidgets with hands or feet or squirms in seat”, is “on the go”, or often acts as if “driven by a motor”, and “Interrupts or intrudes on others (e.g., butts into conversations or games)” were 0.24, 0.31, and 0.33, respectively.

Comparison of total PSQ scores between children reporting different symptom severity and duration demonstrated that PSQ score increased as the symptom severity and duration increased (*p* < 0.001) (Table 2). Total score was calculated to be 0.13 ± 0.11 in children who did not report severe symptoms while it was calculated as 0.4 ± 0.18 in children with apnea during sleep (*p* < 0.001). Similarly, total PSQ score for children that did not report snoring was 0.13 ± 0.11 while that for the ones who snore throughout sleep was 0.32 ± 0.17 (*p* < 0.001).

Table 3 illustrates the sample distribution by residency, gender, and age.

At the completion of the data-entry of all eligible questionnaires, 342 (7.90%) subjects were considered with high risk for SBD, versus 4093 (92.1%) of school children (Table 4). The syndrome prevalence is higher in boys that in girls (8.5% vs. 7.2%) although this difference was not found significant (t = 1.61, *p* value = 0.103).

Table 4 also describes the frequency of SDB risk according to residence status (urban versus rural) where the higher rural prevalence (7.3% vs. 10.4%) is statistically significant (t = 2.86 and *p*-value = 0.004).

Table 5 shows the distribution of SDB prevalence by age, the highest value is at the age of 15 (9.60%) and the lowest at the age of 7 (5.60%). Although the prevalence in various years of age seemed variable, from a statistical point of view there is no significant predisposition to the pathology in one age rather than another (chi-square = 0.92, *p* value > 0.05).

Specifically, Table 6 reports the frequency of subjects at high risk of SDB according to district residence of the sample. The distribution of subjects at high risk for SDB amongst districts varies from a minimum of 4.2% in Elbasan to a maximum of 10.5% in Gjirokastra. There is no statistical association for the geographic distribution of individuals positive to SDB (Chi-Square 9.4, *p*-value > 0.05).

Similarly, the correlation between SDB and overweight/obesity shows a greater frequency of overweighted/obese children and adolescents at risk of SDB (20.8%) compared to normal-weighted ones (6.3%). From the data obtained in Table 7, it is noted that differences between groups is statistically significant (chi-square = 39.7, *p* value < 0.05).

## 4. Discussion

Analyzing SDB, it should be stated that large samples, with more than 1000 subjects, make difficult the employment of both polysomnography and actigraphy. Therefore, in these cases, the questionnaires represent the most valid and easy tool, despite having limitations related to the accuracy of data due to the length of the questionnaire, the level of understanding of the questions, and the actual awareness from the parents on reporting truly required signs and symptoms attributable to SDB. The latest barriers could be limited by a clear described questionnaire to be handed to children caretakers/parents for filling them in. For the same reason, we used questionnaires that we previously tested in the University clinic and that are still applied as a diagnostic tool in the Orthodontics and Pediatric Dentistry Department. As already mentioned, the questionnaire applied in this research has been standardized and it was known its sensitivity and specificity, 81% and 87%, respectively [4]. The pediatric sleep questionnaire represents a valid tool that has previously used in similar studies [18,19]. According to a recent review only this type of questionnaire has shown sufficient diagnostic precision to be used as a screening method for SDB [20].

According to our data we can confirm that the SDRB-PSQ Scale of childhood evaluating snoring, sleepiness, and inattention is a valid and reliable tool. Reliability denotes the ability of a scale to produce consistent results when completed under similar conditions, whereas validity denotes the extent to which a scale measures the construct it is supposed to. Reliability is analogous to the scale’s precision, whereas validity is analogous to its accuracy. Similar results were found for the reliability and validity of Turkish version questionnaire [21]. That can be used to identify SDB or their associated constructs in the search clinical when polysomnography, as a golden standard, is not feasible. Furthermore, this tool identifies the high-risk status for SDB in individuals and not the confirmation the relative diagnose.

The present research concerning the issue of respiratory sleeping problems in growing population is unique in Albania. Early identification and treatment would help to prevent morbidity and complications due to SDB. In the overall sample, out of 4442 individuals, 7.9% are at risk for SDB. This prevalence falls within the range of values found in the literature as evaluated by Lumeng and Chervin, describing the risk of SDB between 4% and 11% [1]. A study conducted by Sanchez et al. has reported a higher prevalence rate (17.7%) of SDB in Chilean schoolchildren [22]. Baidas et al. found that 21% of children in Saudi primary school were at high risk for SDB [23].

According to INSTAT [17], approximately 41.8% of the Albanian population resides in rural areas. In general, the countries tend to have a higher population in urban areas compared to rural ones. This facilitates access to basic services, such health care or education. Consequently, the higher the ratio of urban to rural population, the higher the welfare of the inhabitants of a state. From a statistical point of view, an important significance is detected through the evaluation of prevalence according to urban and rural residence, which shows a greater frequency in the rural areas versus urban (10.4 % versus 7.3%). Probably this is due to the unequal distribution of economic growth and wealth between rural and urban which reflects an increasing un-equality in the health status of the population, as affirmed by National Planning Cycles data (National Institute of Statistics).

This study aims to address this deficiency and to add strength to the evidence that SDB is probably prevalent in the rural population than in the urban areas.

SDB occurs in development individuals of all ages mostly during two peak periods. The first peak prevalence of OSA in children occurs between 2 and 8 years, due to pharyngeal lymphatic tissue hypertrophy [24] A second peak arises during adolescence in relation with weight gain [25]. The prevalence of SDB, distributed per each year of age, was not statistically significant, but the prevalence of individuals at risk of SDB tends to increase in adolescent period. The literature reports a higher risk rate for SDB in males, due to hormonal factors involved [26,27]; however, our study reports SDB affects children and adolescent of both sexes equally [28].

Obesity is a well-established risk factor for developing SDB. It leads to chronic, low-grade inflammation and oxidative stress because of elevated levels of the inflammatory cytokines interleukin-6, tumor necrosis factor alpha, C-reactive protein, and leptin in adolescents with moderate SDB [29,30]. In addition, obesity can increase the risk of upper airway collapse and result in sleep fragmentation, which further increases appetite. leading to weight gain and obesity [31]. Leptin, “the hormone of energy expenditure”, inhibits appetite in the hypothalamus by counteracting the effects of orexigenic hormone neuro-peptide Y and ghrelin. It also enhances the synthesis of appetite suppressant alpha-melanocyte stimulating hormone [32]. Sleep fragmentation attenuates leptin signaling pathways in hypothalamus, leading to an increase in ghrelin to leptin ratio. This leads to uncontrolled appetite and food intake, resulting in weight gain and obesity [33]. Sleep fragmentation disrupts energy homeostasis by inducing endoplasmic reticulum stress and activating unfolded protein response in the hypothalamus, which ultimately results in weight gain [32]. These childhood problems can complicate adulthood, as severe childhood obesity is associated with adulthood obesity. Additionally, the co-morbidities of severe obesity can have lasting sequelae, such as cardiovascular, metabolic, and neurocognitive morbidities [34].

The present study also reveals that the prevalence of SDB considerably increases from 6.3% in the normal-weighted population to 20.8% in the population having BMI ≥ 25 kg/m^2^, which means every other overweight/obese participant is at risk of having SDB. A similar result has been obtained by another study by Kohler [35], who demonstrated that the risk of SDB increased 3.5 times to obese children in comparison with non-obese ones. Even though adenotonsillectomy represents the first line of therapy for these children, several studies have reported that obesity increases the risk of persistent OSAS after surgery. Weight-loss management could be the key in the treatment of obesity-related OSAS in children and adolescents, especially in those that have previously performed adenotonsillectomy surgery without clinical results [36].

*Study limitations*. The risk of SDB was subjectively determined using Pediatric Sleep Questionnaire. Accuracy of SDB with attended or unattended overnight polysomnography is important to determine the prevalence and the severity of SDB. Furthermore, in the study population, only 72.98% of original sample was considered eligible data.

## 5. Conclusions

The present study highlights a high percentage of growing subjects at risk for respiratory sleep disorders (7.9%), with a major prevalence in Albanian rural areas. The lack of national guidelines for prevention in general [37] and in treatment of SDB reflects the absence of awareness for this pathology amongst health specialists and in the general public. In this specific context, the orthodontists should take a crucial role due to their interception about oro-cranio-facial district during developmental age. Even if, in this study, the risk of SDB was determined using the Pediatric Sleep Questionnaire, it is a first epidemiological evaluation in Albanian growing population, in a country that needs to improve the prevention about SDB.

The orthodontist takes a fundamental position within the medical team, since a careful evaluation of craniofacial growth, occlusion, weight, the typical clinical appearance, and radiographic examinations could be applied jointly with the questionnaire concluding a diagnosis of SDB, as already highlighted in other studies reported in the literature [1].

## Figures and Tables

**Table 1:** Internal consistency of the PSQ (Cronbach alpha values).

Internal Consistency of the PSQ	
Domain	Cronbach’s Alpha
Snoring domain	0.63
Sleepiness domain	0.38
Inattention domain	0.53

**Table 2:** Comparison of PSQ scores between different symptom groups.

Type of Symptom		PSQ Scores			
		Total	Snoring	Sleepiness	Inattention
Symptom Severity	None	0.13 ± 0.11	0.02 ± 0.09	0.2 ± 0.25	0.23 ± 0.25
	Loud snore	0.35 ± 0.17	0.7 ± 0.23	0.29 ± 0.27	0.33 ± 0.30
	Difficulty breathing	0.33 ± 0.16	0.27 ± 0.34	0.29 ± 0.28	0.38 ± 0.29
	Apnea during sleep	0.4 ± 0.18	0.32 ± 0.38	0.32 ± 0.27	0.47 ± 0.31
*p* value		<0.00	<0.00	<0.00	<0.00
Symptom duration	Never	0.13 ± 0.11	0.01 ± 0.07	0.2 ± 0.25	0.23 ± 0.25
	Snore > half of sleep	0.31 ± 0.15	0.57 ± 0.26	0.3 ± 0.27	0.31 ± 0.27
	Snore throughout sleep	0.32 ± 0.17	0.68 ± 0.26	0.26 ± 0.27	0.32 ± 0.29
*p* value		<0.00	<0.00	<0.00	<0.00

**Table 3:** Descriptive data of studied sample by residence, sex and age.

District	Total Sample	Residence		Sex		Age (in years)		
		Urban	Rural	Male	Female	6–9	10–12	13–15
Elbasani	24	24	0	5	19	6	5	13
Tirana	1869	1388	481	880	989	810	695	364
Durrësi	829	548	281	392	437	286	350	193
Gjirokastra	172	172	0	82	90	64	63	45
Saranda	171	171	0	76	95	65	88	18
Dibra	264	264	0	138	126	98	100	66
Bulqiza	248	248	0	130	118	76	140	32
Shkodra	526	526	0	263	263	215	206	105
Vlora	339	339	0	154	185	115	130	94
Total	4442	3680	762	2120	2322	1735	1777	930

**Table 4:** Frequency of subjects with high risk SBD (gender and residence status). * Confidence Interval.

	Subjects at Risk of SBD		Gender		Residence Status	
	No	Yes	Male	Female	Urban	Rural
Number	4093	349	2120	2322	3680	762
Prevalence	92.10%	7.90%	8.50%	7.20%	7.30%	10.40%
CI * (%)		7.1 to 8.7%	7.3 to 9.7%	6.1 to 8.3%	6.5 to 8.1%	8.2 to 12.6%

* Confidence Interval.

**Table 5:** Frequency of subjects with high risk SBD by age.

Age (in years)	Number	Prevalence	CI (%)
6	83	8.40%	2.4 to 14.4%
7	445	5.60%	3.5 to 7.7%
8	536	6.00%	4.0 to 8.0%
9	671	7.90%	5.9 to 9.9%
10	557	9.00%	6.6 to 11.4%
11	634	9.00%	6.8 to 11.2%
12	586	7.50%	5.4 to 9.6%
13	425	8.50%	5.8 to 11.2%
14	287	8.40%	5.2 to 11.6%
15	218	9.60%	5.7 to 13.5%

**Table 6:** Frequency of subjects with high risk SBD per district.

District	Number	Prevalence	CI (%)
Elbasani	24	4.20%	0 to 12.2%
Tirana	1869	8.00%	6.8 to 9.2%
Durrësi	829	7.40%	5.6 to 9.2%
Gjirokastra	172	10.50%	5.9 to 15.1%
Saranda	171	7.00%	3.2 to 10.8%
Dibra	264	7.20%	4.1 to 10.3%
Bulqiza	248	10.90%	7.0 to 14.8%
Shkodra	526	5.90%	3.9 to 7.9%
Vlora	339	9.10%	6.0 to 12.2%
All sample	4442	7.90%	7.1 to 8.7%

**Table 7:** Frequency of subjects with high risk SBD per obesity.

BMI ≥ 25	SBD				
	Value	YES	%	No	%
Yes	400	83	20.8	317	79.2
No	3938	250	6.3	3688	93.7
Don’t know	65	12	18.4	53	81.6
No answer	39	6	15.3	33	84.7
Total	4442	351	7.9	4091	92.1
